# Steady state vascular imaging with extracellular gadobutrol: evaluation of the additional diagnostic benefit in patients who have undergone a peripheral magnetic resonance angiography protocol

**DOI:** 10.1186/1532-429X-15-97

**Published:** 2013-10-25

**Authors:** Melissa M Ong, Katharina Hausotter, Lothar R Pilz, Stefan O Schoenberg, Henrik J Michaely

**Affiliations:** 1 Institute of Clinical Radiology and Nuclear Medicine, Medical Faculty Mannheim, University of Heidelberg, Theodor-Kutzer-Ufer 1-3, 68167 Mannheim, Germany; 2 Medical Faculty Mannheim, University of Heidelberg, Theodor-Kutzer-Ufer 1-3, 68167 Mannheim, Germany

**Keywords:** Steady state, Magnetic resonance angiography, Vascular imaging, Venous imaging, Gadobutrol, 3T

## Abstract

**Background:**

To evaluate the feasibility and additional diagnostic benefit of a high-resolution steady state 3D-volume interpolated breath-hold exam (VIBE) sequence between a continuous table movement (CTM) MR angiography of the entire runoff vasculature and a time-resolved (TWIST) MRA of the calves.

**Methods:**

In this retrospective IRB approved study 224 patients (72 women, 152 men, mean age 67.29 ± 13.9) were included who had undergone a low-dose MR angiographic protocol at 3T (Siemens TimTrio) after injection of 0.1 mmol/kg gadobutrol including a CTM MRA, a time-resolved MRA of the calf station and a steady state 3D VIBE sequence prior to the time-resolved MRA. One board-certified radiologist rated the image quality of the steady state VIBE sequences on an ordinal three point scale (excellent, good, poor) and analyzed the images for additional diagnostic findings of and beyond the vascular system in comparison to the CTM MRA and the time-resolved MRA. Descriptive statistics and demographic patient data were used for further evaluation.

**Results:**

The image quality of the steady state imaging of the pelvis, upper and lower leg was excellent in up to 88%, 84% and 47%, respectively, while poor image quality was only detected in the upper (2%) and lower leg (6%). An additional diagnostic benefit was found in 44% of the patients overall. The most common relevant pathologies included inflammatory processes of the soft tissues (26%), thrombi (14%), abscesses (13%) and tumors (11%). In subgroups of patients above the age of 60, 65, 70, 75 and 80 years an additional pathology was found in 50% 33%, 44%, 65% and 58%, respectively. There was no significant difference in terms of additional findings between men and women (46% and 39%, p > 0.05) and inpatients and outpatients (42% and 45%, p > 0.05).

**Conclusion:**

Steady state imaging is also feasible with extracellular contrast agents with good image quality yielding additional diagnostic findings in up to 44% and above in patients older than 60 years of age irrespective of gender or patient status. Given the short acquisition time of 4 minutes this sequence could be added to all peripheral MRA exams.

## Background

Several imaging modalities allow for the depiction of the vascular anatomy of patients with peripheral arterial occlusive disease (PAOD). Digital subtraction angiography (DSA) has been the gold standard method [1], but it involves iodinated and potentially nephrotoxic contrast agents as well as ionizing radiation and the accompanied risks. In addition, DSA is expensive and invasive compared to other imaging methods. Significant complications such as hemorrhage or emboli following peripheral DSA occur in approximately 2% [2].

In contrast, computed tomography angiography (CTA) is a minimally invasive imaging method with a shorter examination time while maintaining a high diagnostic accuracy [3]. As in DSA, CTA is also limited by potentially nephrotoxic contrast agents, decreased diagnostic accuracy in heavily calcified vessels, use of ionizing radiation and its limited soft tissue contrast [4].

Peripheral contrast enhanced (CE)-MRA has been proven as the method of choice for non-invasive imaging of the vasculature without radiation exposure or nephrotoxic contrast agents while maintaining high diagnostic accuracy [5]. The basic technique employed for peripheral CE-MRA is the bolus-chase method which involves intravenous injection of a paramagnetic MR contrast agent and acquisition of T1-weighted images in the subsequent arterial first-pass phase [6]. In order to achieve high quality MR angiograms without interfering venous contamination or artifacts optimization of peripheral CE-MRA by implementing hybrid scan protocols have been proposed and proven to be feasible [7-9]. This involves a separate injection of contrast media to image the calf region for a time-resolved MRA to safely depict this critical vascular territory and to increase the diagnostic accuracy [10].

At our site a low-dose MR angiographic protocol that consists of a continuous table movement MRA (CTM-MRA) of the entire runoff vasculature with 0.07 mmol/kg Gd-chelate and a time-resolved MRA with stochastic interleaved trajectories (TWIST-MRA) of the calf station with 0.03 mmol/kg Gd-chelate as described in the literature is being followed [8]. Additionally, a high-resolution steady state 3D-volume interpolated breath-hold exam sequence was introduced prior to the time-resolved MRA with two aims: make positive use of the time delay between the CTM-MRA and the TWIST-MRA and to be able to assess extra-vascular and venous structures more closely. Steady-state imaging is a concept in CE-MRA that offers depiction of both arteries and veins – yet so far, steady state imaging has only been performed after the application of intravascular contrast agents such as gadofosveset [11-13].

Up until now there has been no research pursued regarding the feasibility of steady state imaging with injection of gadobutrol – an extracellular contrast agent – and the additional vascular and non-vascular pathology found in this kind of imaging. Therefore, the aim of this study was to assess the feasibility of such imaging and to investigate if specific subgroups of patients show a greater benefit from steady state imaging.

## Methods

This retrospective single-center study was pursued without any financial support from industry. The authors had complete control of the data submitted for publication. This study was approved by our local ethics committee (medical ethics committee II, University Medicine Mannheim, Ruprecht-Karls-University Heidelberg), reference number 2008-338N-MA and pursued in compliance with the Helsinki Declaration. Due to its retrospective nature of our study informed consent has been waived and all data has been analysed anonymously.

### Patients

Two hundred and twenty-four consecutive patients who had undergone a peripheral MRA exam between January 2010 and May 2012 were included in this retrospective study. The characteristics of the study population are listed in Table 1.

**Table 1:** Characteristics of the study population

Patient count	224
Mean age (years)	67.29
SD (years)	± 13.9
Age range (years)	15 - 95
Men	152
Women	72

Further analysis included extraction of the patients clinical information out of the clinical information system *(ISH-Med, SAP, Walldorf, Germany)* concerning the following risk factors: hypertension, hyperlipidemia, smoking and diabetes.

### MR angiography

**Figure 1:**
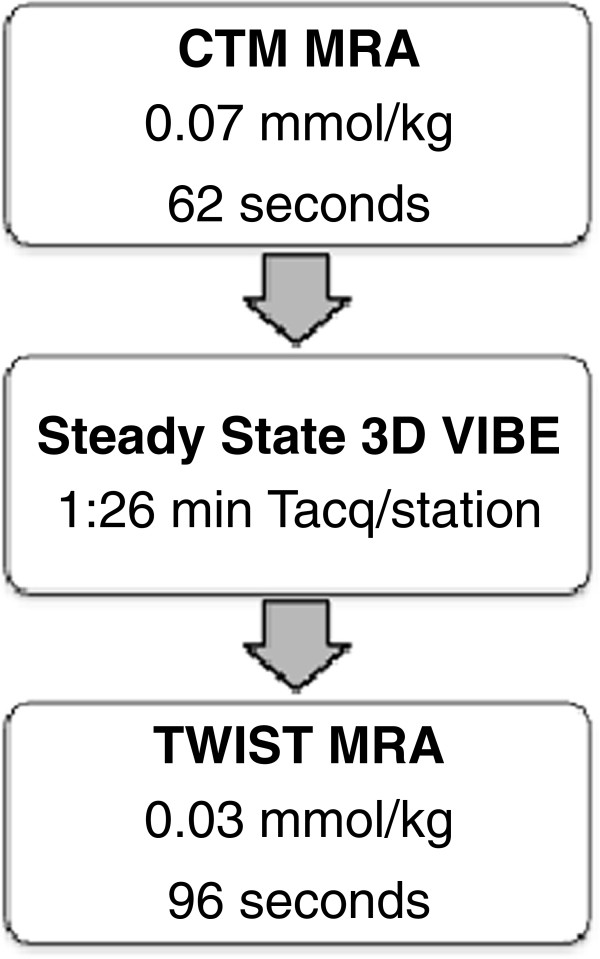
Graphic depiction of the MRA protocol and the order of the acquisition.

**Figure 2:**
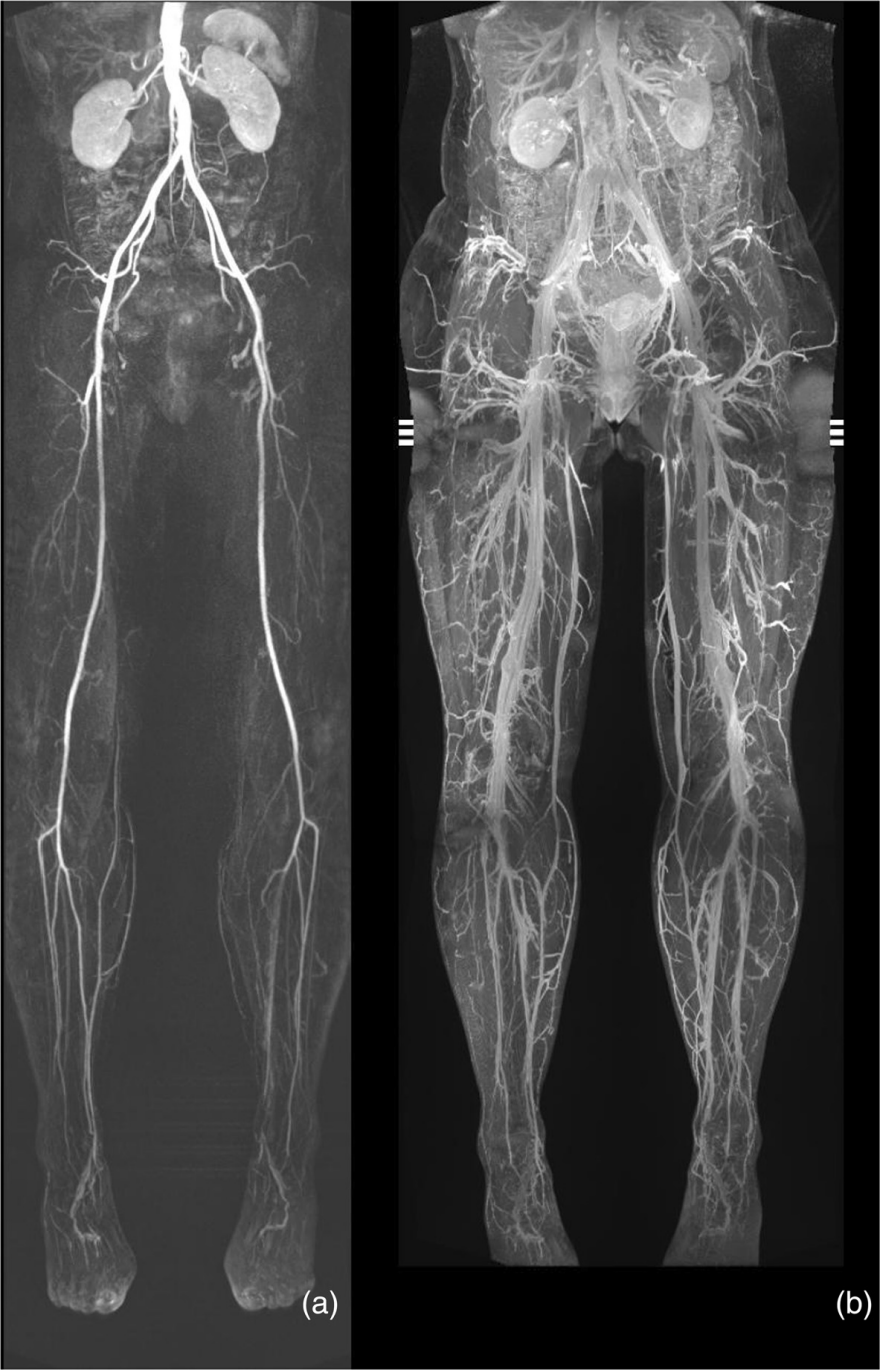
**Coronal MIP-images of a 44-year-old woman without any pathologies. (a)** Conventional MRA. **(b)** Steady-state 3D VIBE sequence

MRA examinations were performed on a 3.0T MR scanner (Magnetom TimTrio; Siemens Healthcare Sector, Erlangen, Germany) using a dedicated matrix coil with 36 independent coil elements and two 6-element body matrix coils for signal reception in addition to two clusters of the built-in 32-element spine matrix. Patients were positioned feet first supine. The low-dose MRA protocol consisted of a CTM-MRA (TR/TE, 2.4/1.0 ms; 21° flip angle; 1.2 mm isotropic; gadolinium dose 0.07 mmol/kg BW), a time-resolved (TWIST) MRA of the calf station (TR/TE 2.8/1.1 ms; 20° flip angle; 1.1 mm isotropic; 5.5s temporal resolution; gadolinium dose 0.03 mmol/kg BW) and a steady state 3D VIBE sequence (TR/TE 3.8/1.2 ms; 12.5° flip angle; 1.3 mm isotropic; PAT 3) prior to the time-resolved MRA. The steady state MRA was acquired in three steps (pelvis, thighs, calves) and composed afterwards to yield a single large field of view. The timing of the sequences is shown in Figure 1. An example of coronal MIP-images of a CTM MRA and a steady state 3D VIBE sequence of a healthy woman without any pathologies is shown in Figure 2.

### Contrast agent

For this study gadobutrol (1.0 M Gadovist®; Bayer Healthcare, Berlin, Germany), a gadolinium-based, macrocyclic, paramagnetic contrast agent was administered in the abovementioned protocol. A cumulative dose of 0.1 mmol/kg (1.0 mol/L; 0.07 mmol/kg for CTM MRA, 0.03 mmol/kg for TWIST MRA) was used. For equalization of injection volumes gadobutrol was diluted with saline in a 1:1 ratio. Contrast agent was injected at a rate of 1.5 ml/sec via an 18-gauge needle in the left or right cubital vein by using an automated power injector (Spectris Solaris EP; Medrad, Indianola, PA). Subsequently, an injection of a 30-mL saline bolus was administered at the same injection rate.

### Image analysis of MRA

One board-certified radiologist (blinded, >10 years of experience in vascular imaging) performed image analysis of the steady state MRA including pelvis, upper and lower leg regions offline with an OsiriX DICOM viewer 3.8.1 (OsiriX Foundation, Geneva, Switzerland). Overall image quality was analyzed on a three point scale (3: excellent image quality allowing assessment of arteries, vessels and soft tissue; 2: good image quality allowing impaired assessment of arteries, vessels and soft tissue; 1: poor image quality not allowing sufficient assessment of the vascular and/or soft tissue structures). The presence of additional diagnostic findings of the vascular system and beyond was noted. All findings which could not be seen on either the CTM-MRA or the TWIST-MRA were considered to represent additional findings.

### Statistical analysis

Descriptive statistics (median, mean, 95% confidence interval and standard deviation (SD)) were used for all data including evaluation of overall image quality, additional diagnostic findings, as well as patient status and demographic patient data. A Wilcoxon rank sum test was used to analyze a relation between age and additional pathology. Relationship between specific risk factors and additional pathology was analyzed by chi-square test. The results were considered statistically significant if the *p* value was no greater than 0.05. SAS statistical software (SAS 9.2 TS Level 2M3, 2012, SAS Institute Inc., Cary, NC, USA) was used for statistical analysis.

## Results

### Image quality

Steady state imaging was successfully performed in all patients. Image quality in the pelvic region was rated as excellent in 187 (88%) of 212 cases, good in 24 (11%) cases and poor in one case. Further results showed 185 (84%) out of 222 with excellent, 33 (15%) with good and 4 (2%) with poor image quality for the upper leg, as well as 104 (47%) out of 220 with excellent, 102 (46%) with good and 14 (6%) with poor image quality ratings for the lower leg (Table 2). The main reason for image degradation were motion and particularly for the calves insufficient spatial resolution to clearly separate the arteries from the veins. 12 data-sets for the pelvic region, 2 for the upper leg region and 4 for the lower leg region were not analyzed due to missing data in the PACS.

**Table 2:** Results of image analysis

**Image quality score**	**Pelvis**	**%**	**Upper leg**	**%**	**Lower leg**	**%**
3	187	88.2	185	84	104	47.3
2	24	11.3	33	14.8	102	46.4
1	1	0.5	4	1.8	14	6.4
Total	212	100	222	100	220	100

### Pathologies

**Figure 3:**
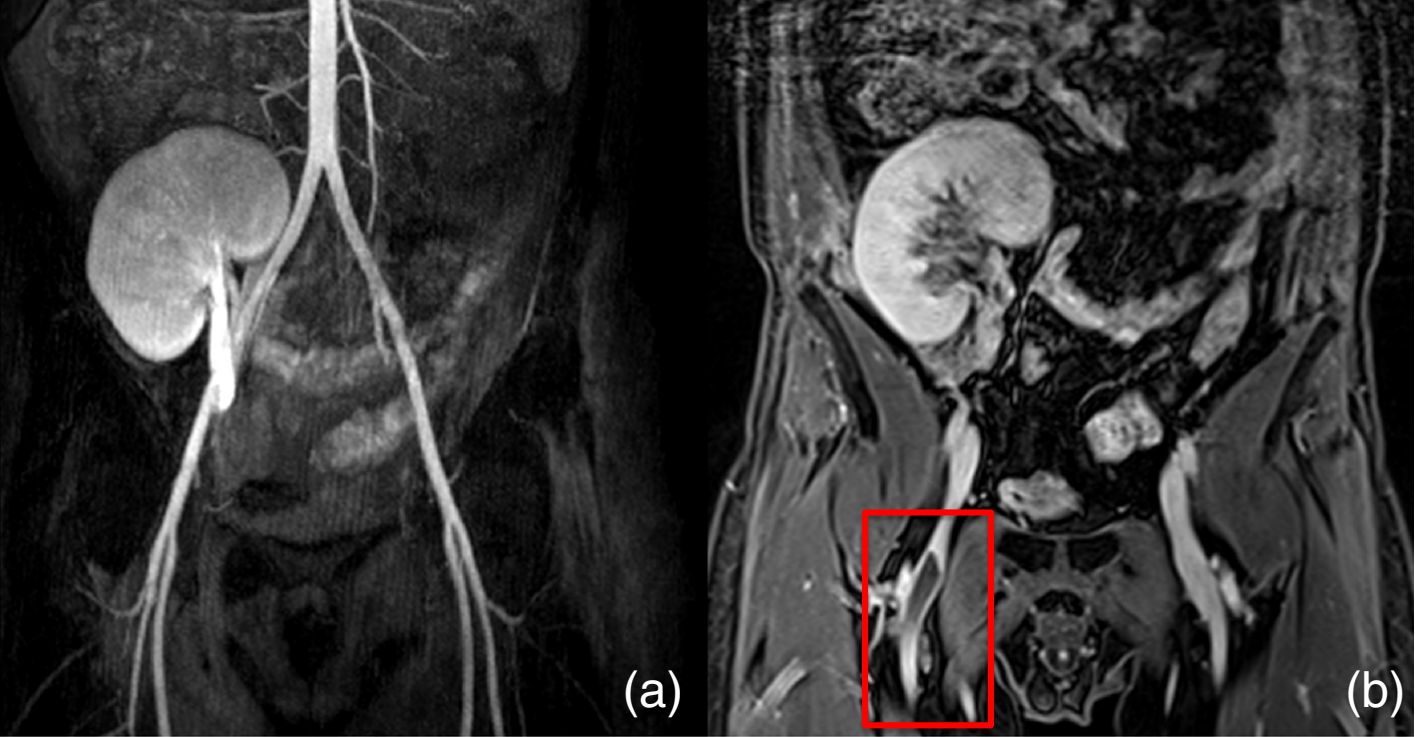
**Coronal images of a 42-year-old man. (a)** Conventional MRA. **(b)** Steady-state 3D VIBE sequence showing a thrombus in the right superior femoral vein (box).

**Figure 4:**
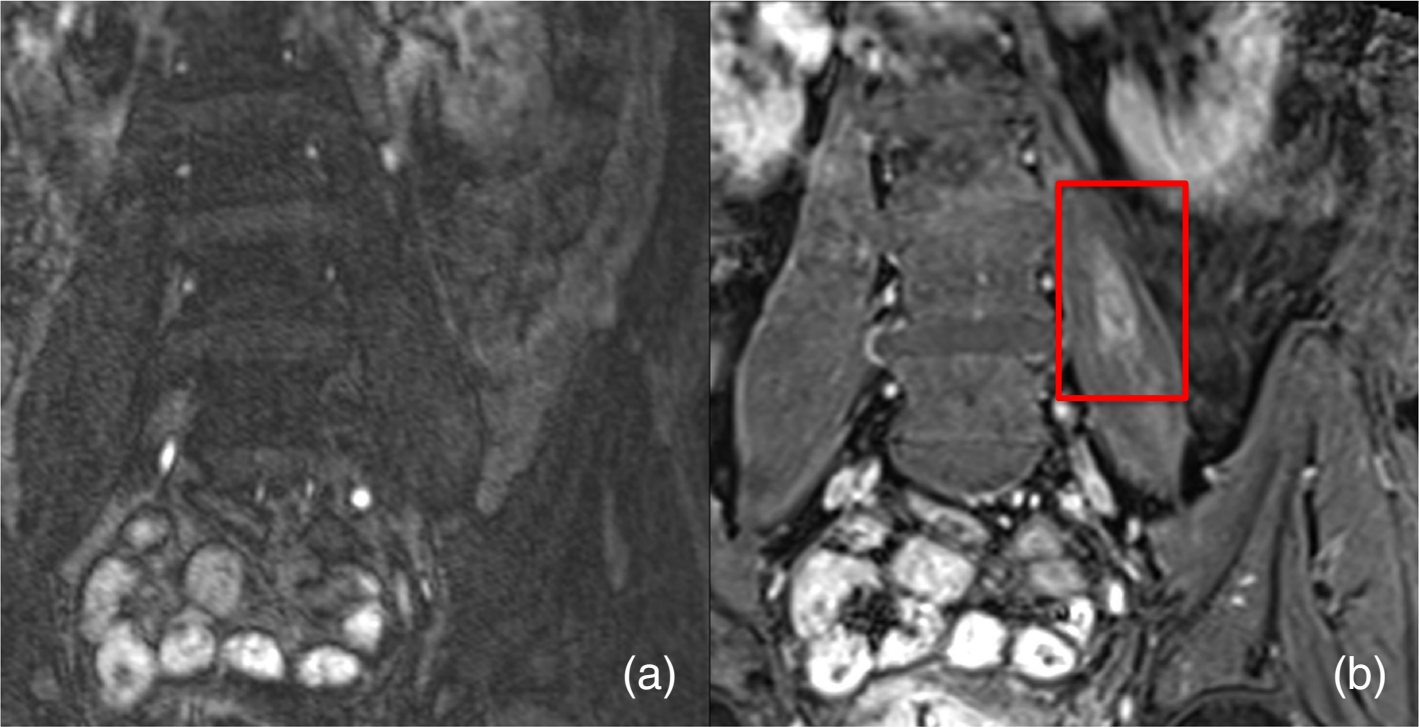
**Coronal images of a 69-year-old man. (a)** Conventional MRA. **(b)** Steady-state 3D VIBE sequence showing an abscess in the left psoas muscle (box).

**Figure 5:**
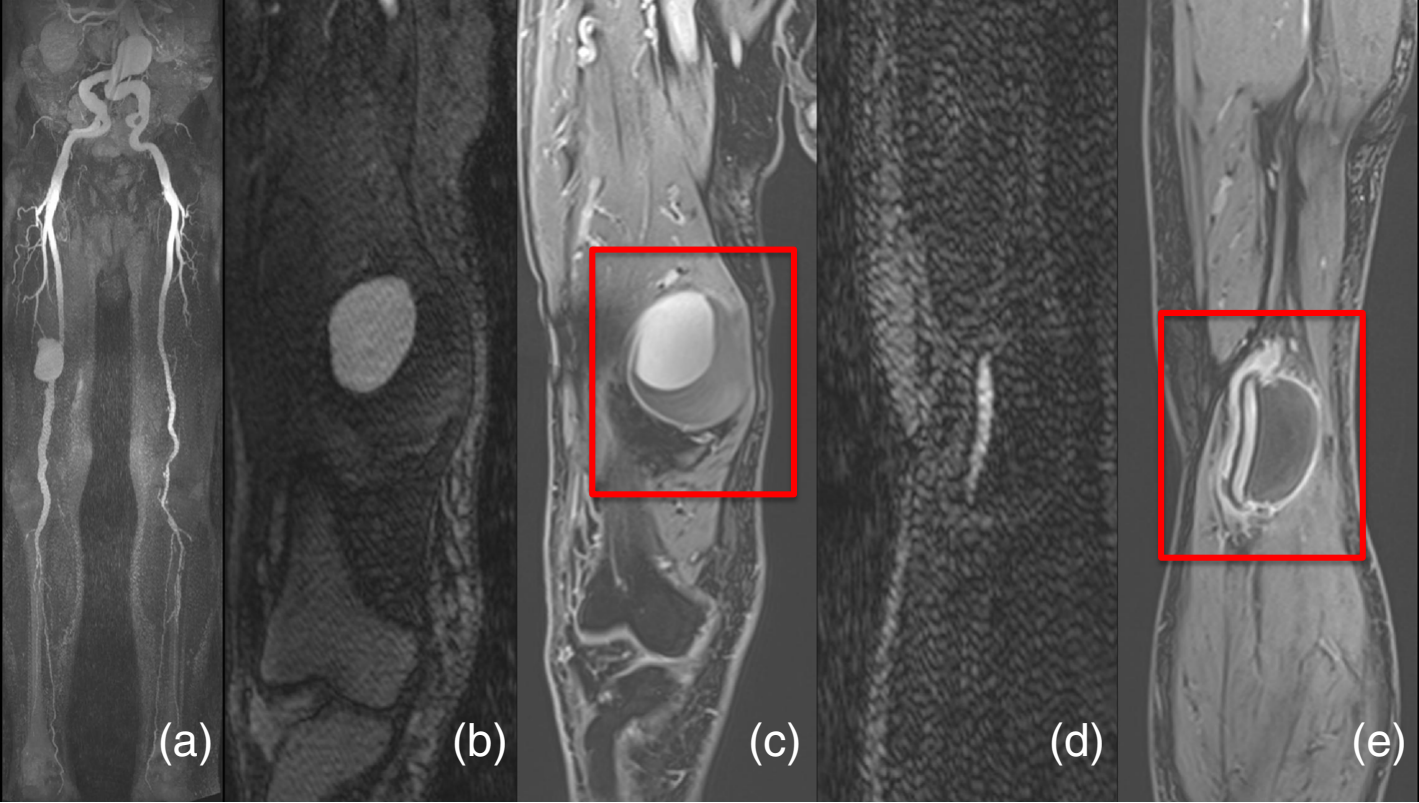
**Coronal images of an 84-year-old man. (a, b, d)** Conventional MRA. **(c, e)** Steady-state 3D VIBE sequence showing a non-contrast-enhanced aneurysm (box).

Osteochondrosis, joint and muscle infections were included under inflammatory processes, which shared 26% of the additionally found pathologies. Bursitis of the trochanter was detected in 17%, varicosis and thrombi (Figure 3) in 14% each, abscesses (Figure 4) in 13%, tumors in 11% (e.g. osseous metastases, bladder cancer) and synovitis of the knee in 9%. Further pathologies which accounted for less than 5% included wall thickening of the sigmoid, arterial emboli, arterial aneurysms (Figure 5), varicoceles, phlegmons and occluded bypasses.

### Age

**Figure 6:**
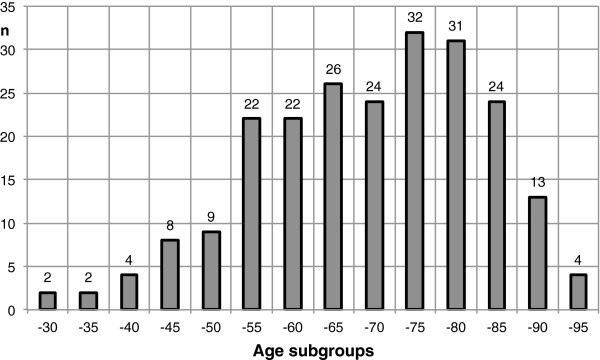
Age distribution of the study population.

In the subgroup of patients with the age of up to 60 years additional pathology could be found in 50%, in the subgroups with the age up to 70, 75, 80, 85 and 90, additional pathology was found in 33%, 44%, 65%, 58% and 46%, respectively. The age distribution of the patient cohort is shown in Figure 6.

### Gender

No significant difference with respect to additional pathology could be found based on gender using a chi-square test: 70 (46%) out of a total of 152 male patients compared to 28 (39%) out of 72 female patients showed an additional pathology in the added steady state 3D-VIBE sequence (p > 0.05) (Table 3).

**Table 3:** Results of gender analysis

	**Additional pathology**	**No additional pathology**	**Total**	**%**
Male	70	82	152	68
Female	28	44	72	32
Total	98	126	224	100

### Patient status

No significant difference with respect to additional pathology could be found based on patient status using a chi-square test: 41 (45%) out of a total of 91 outpatients compared to 56 (42%) out of 132 inpatients patients showed an additional pathology in the added steady state 3D-VIBE sequence (p > 0.05) (Table 4).

**Table 4:** Results of patient status analysis

	**Additional pathology**	**No additional pathology**	**Total**	**%**
Outpatient	41	50	91	41
Inpatient	56	76	132	59
Total	97	126	223	100

### Risk factors

**Figure 7:**
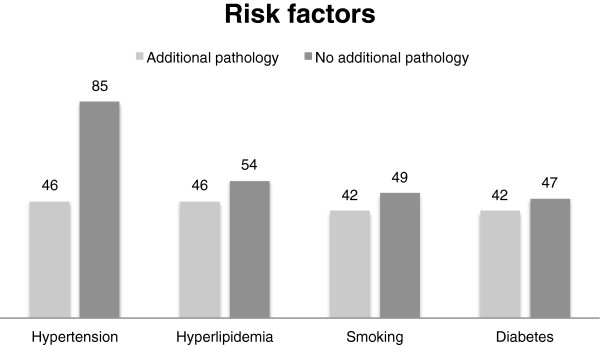
Patients with the following risk factors of peripheral arterial occlusive disease (PAOD): hypertension, hyperlipidemia, smoking and diabetes respective to showing an additional pathology in the added steady-state-VIBE sequence (%).

Patient data was analyzed concerning risk factors for peripheral arterial occlusive disease. Out of the total of 224 patients 195 had sufficient clinical information about risk factors including hypertension, hyperlipidemia, smoking and diabetes. 85% of those patients suffered from hypertension, whereas 54% suffered from hyperlipidemia. 46% in each group showed relevant additional pathologies in the steady state VIBE sequence. 49% and 47% of the patient cohort smoked and suffered from diabetes, respectively. 42% in each group (smoking/diabetes) showed relevant additional pathologies (Figure 7). Statistical analysis could not show any significant relation between risk factors and additional pathologies.

**Figure 8:**
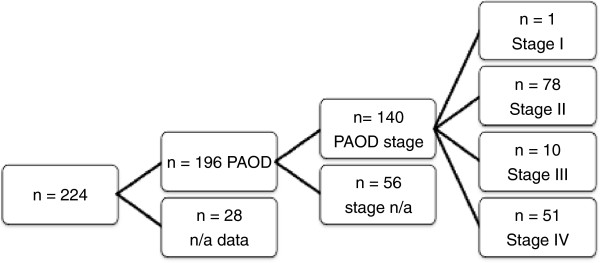
Flow diagram showing the number of patients with specific stages of peripheral arterial occlusive disease (PAOD).

Patient data confirming PAOD existed for 196 patients. For 140 patients information about the specific PAOD-stage was available. With a total of 78 patients stage II was represented the most, followed by 51 patients classified as stage IV, 10 patients as stage III and merely one patient as stage I (Figure 8).

## Discussion

Over the past 5 years MRA has undergone substantial improvements including high image quality and high diagnostic accuracy at low amounts of Gd-chelate [14,15]. For patients with peripheral arterial occlusive disease (PAOD) contrast-enhanced MRA has become the most appropriate noninvasive diagnostic method of choice [16]. Systematic reviews have confirmed the high diagnostic accuracy of peripheral CE-MRA [5].

Conventional peripheral CE-MRA is based on a bolus-chase method which involves imaging over three stations from the aortic bifurcation to the periphery after single-injection of contrast media. This may result in venous contamination of the lower leg region particularly in patients with higher stages of PAOD and concomitant risk factors [17]. To overcome venous contamination in order to maintain high diagnostic accuracy for every station, new techniques have been developed such as the hybrid scan protocol which can be performed by two different ways: a large field-of-view MRA after the first injection of contrast agent followed by a time-resolved (TWIST) MRA of the calf station after the second injection or by scanning the peripheral region first followed by the pelvic/thigh region after the second injection of contrast media [9,18]. Both techniques have specific advantages but both have a time delay between the two contrast agent injections in common. This time delay was filled with the acquisition of a steady state MRA at our institution aiming at the detection of additional – particularly extra-vascular – pathology. Due to the time lag between the CTM and TWIST MRA there is almost no significant time loss by implementing the steady state VIBE sequence. Up until now, there has been no research pursued concerning the impact and occurrence of additional pathology in steady state imaging: initial steady state imaging with intravascular contrast agents was not suited for the detection of extra-vascular pathologies and steady state imaging with extracellular contrast agents has not been attempted or at least not been published. This led to initiating the present retrospective study.

This study has proven that by implementing a steady state VIBE sequence in a routine MRA, relevant additional pathology can be acquired with good image quality that allows identification of additional pathologic findings without added injection of contrast media and with almost no time loss. In contrast to our initial assumptions, the presence of additional pathology was found irrespective of gender or patient status. We evaluated the incidence of risk factors for PAOD such as hypertension, hyperlipidemia, smoking and diabetes [19,20]. The results of our study could not show an increased number of additional pathologies in patients with the aforementioned risk factors for PAOD. There was no difference in the incidence of additional pathology between inpatients and outpatients either. This was unanticipated since a higher incidence of pathologies was expected in inpatients. The additional pathologies included a wide range of diseases: from less important findings such as bursitis (17%) to relevant pathologies that required medical attention and treatment such as deep venous thrombi (14%), soft tissue abscesses (13%) and malignant tumors (11%).

Our findings concur with a previous publication that has used the intravascular contrast agent gadofosveset which thus implicate a blood pool role for extracellular contrast agents such as Gadobutrol. Hadizadeh et. al have investigated the prevalence of incidental deep venous thrombosis (DVT) in patients with clinically suspected PAOD who had undergone CE-MRA on a 1.5T scanner. Similar to our sample size 245 patients were included. Incidental DVT was observed in 11%, comparable to our results with 14% of incidental thrombi [21]. The image quality of the steady state VIBE images tended to be worse at the calf station in our study. This was due to the limited spatial resolution that did not always allow separating arteries and veins. A previous study using gadofosveset demonstrated that a spatial resolution of 0.5 mm isotropic was necessary to safely identify arterial and venous structures [13].

### Study limitations

Since this study was conducted as a retrospective analysis of peripheral MRA at our institution not all of the clinical information for each patient could be retrieved. The influence of additionally found pathologies with regard to possible changes in patient care has not yet been analyzed. However, the main goal of our study was to quantify additional pathology found, as well as analysis regarding patient characteristics including risk factors. Therefore, further investigation concerning therapeutic management changes have not been part of this study. Future studies will be needed to assess the true clinical value of applying steady state VIBE sequences in patients who undergo a peripheral MRA. Given that all of the examinations were scanned on a 3T scanner where the contrast agent efficacy is higher than at 1.5T results may not be transferable to a 1.5T scanner [22]. Theoretically, the protocol used for this study can be implemented on a 1.5T scanner.

## Conclusion

Our study has shown that steady state 3D VIBE imaging with extracellular contrast agents is feasible with good image quality and additional diagnostic findings in 44% and above in patients older than 60 years irrespective of gender or patient status.

Without additional contrast media application or significant time loss, steady state imaging may further improve the diagnostic value of peripheral MRA.

## Competing interests

The authors declare that they have no competing interest.

## Authors’ contributions

KH carried out the data acquisition. LRP performed the statistical analysis. MMO participated in the design of the study, contributed to the statistical analysis and drafted the manuscript. HJM participated in the design of the study, has made substantial contributions to interpretation of data and revised the manuscript. HJM and SOS have given final approval of the version to be published. All authors read and approved the final manuscript.
